# Mitochondrial respiration in peripheral arterial disease depends on stage severity

**DOI:** 10.1111/jcmm.18126

**Published:** 2024-03-27

**Authors:** Fiona Speichinger, Alexandra Gratl, Ben Raude, Larissa Schawe, Jan Carstens, Nina A. Hering, Andreas Greiner, Dominik Pesta, Jan Paul Frese

**Affiliations:** ^1^ Department of Vascular Surgery Charité Universitätsmedizin Berlin Berlin Germany; ^2^ Department of General and Visceral Surgery Charité Universitätsmedizin Berlin Berlin Germany; ^3^ Department of Vascular Surgery Medical University of Innsbruck Innsbruck Austria; ^4^ Institute of Aerospace Medicine German Aerospace Center (DLR) Cologne Germany; ^5^ Centre for Endocrinology, Diabetes and Preventive Medicine (CEDP) University Hospital Cologne Cologne Germany; ^6^ Cologne Excellence Cluster on Cellular Stress Responses in Aging‐Associated Diseases (CECAD) Cologne Germany

**Keywords:** chronic limb‐threatening ischemia, intermittent claudication, mitochondrial function, mitochondrial respiration, myopathy, PAD, peripheral arterial disease

## Abstract

Peripheral arterial disease (PAD) is an increasing cause of morbidity and its severity is graded based on clinical manifestation. To investigate the influence of the different stages on myopathy of ischemic muscle we analysed severity‐dependent effects of mitochondrial respiration in PAD. Eighteen patients with severe PAD, defined as chronic limb‐threatening ischemia, 47 patients with intermittent claudication (IC) and 22 non‐ischemic controls were analysed. High‐resolution respirometry (HRR) was performed on muscle biopsies of gastrocnemius and vastus lateralis muscle of patients in different PAD stages to investigate different respiratory states. Results from HRR are given as median and interquartile range and were normalized to citrate synthase activity (CSA), a marker for mitochondrial content. In order to account for inter‐individual differences between patients and controls, we calculated the ratio of O₂‐flux in gastrocnemius muscle over vastus muscle (‘GV ratio’). CSA of the gastrocnemius muscle as a proxy for mitochondrial content was significantly lower in critical ischemia compared to controls. Mitochondrial respiration normalized to CSA was higher in IC compared to controls. Likewise, the GV ratio was significantly higher in IC compared to control. Mitochondrial respiration and CSA of PAD patients showed stage‐dependent modifications with greater changes in the mild PAD stage group (IC).

## 1 INTRODUCTION

More than 236 million people worldwide are suffering from peripheral arterial disease (PAD), with increasing incidence owing to an aging population.^1^ Well‐known cardiovascular risk factors foster the development of chronic atherosclerotic disease in peripheral arteries.^2^ According to the clinical presentation of the patient, PAD is divided into defined stages of severity. In the milder stage of intermittent claudication (IC), or Grade 1 according to the Rutherford classification for chronic limb ischemia,^3^ the patient is suffering from exercise‐dependent pain in the affected leg, which resolves when resting. Chronic limb‐threatening ischemia (CLTI) as the most severe stage of PAD is associated with a high amputation rate.^4^ Clinical guidelines define CLTI by the presence of at least one of the following criteria: ischemic rest pain, ischemic trophic lesions, ankle brachial pressure index (ABPI) of less than 0.4, toe pressure of <30 mmHg and/or transcutaneous oxygen saturation (tcPO_2_) of <10 mmHg.^5^ PAD induces chronic ischemia by stenosis or occlusion of the supplying arteries of the lower extremities, resulting in decreased blood flow and lack of oxygen and nutrient supply. Repeated cycles of ischemia and reperfusion of the affected skeletal muscles induce myopathy, with alterations of the mitochondrial respiratory chain playing a crucial role in the pathophysiology of PAD.^6^ In addition, recent multiomics analyses revealed differentially expressed pathways in PAD that may be associated with functional impairment of the patients.^7^, ^8^ Besides morphological changes on the level of the myofibrils,^9^ PAD results in alterations of mitochondrial function, which can be determined by Clark electrode^10^ or high‐resolution respirometry.^11^, ^12^ More specifically, analysis of the respiratory chain revealed alterations in enzymatic activity and mitochondrial respiration of complexes I, III and IV.^13^ In addition, biomarkers for oxidative stress are increased, and antioxidative capacity is decreased.^13^ Alteration of the mitochondrial respiratory chain can therefore contribute to oxidative stress.^14^ Whereas Ryan et al.^15^ only showed alteration of the CLTI group and concluded a unique mitochondriopathy of higher PAD stages, another group showed preserved mitochondrial respiration in PAD.^16^ In summary, while some authors describe altered mitochondrial function, content and oxidative stress levels in patients with peripheral arterial disease^10^, ^13^, ^15^, ^17^ others show preserved mitochondrial respiratory function in this population.^16^ These differences are likely related to differences in disease severity, small sample sizes or heterogenous inclusion criteria. High‐resolution respirometry (HRR) enables standardized in vitro investigation of mitochondrial respiration under biochemically controlled conditions and saturating oxygen supply.^18^ In order to account for mitochondrial content within evaluated muscle samples, respiration can be normalized by citrate synthase activity (CSA), a mitochondrial marker enzyme.^19^

In a previous study involving a small number of IC patients with isolated lesions of the superficial femoral artery, we demonstrated an increased oxygen consumption in biopsy samples of the m. gastrocnemius as a compensatory mechanism in a mild stage of PAD.^11^, ^12^ Other research groups found a decrease in mitochondrial respiration, which may be due to the clinical heterogeneity in the cohort of PAD patients.^10^, ^13^, ^15^, ^17^ Therefore, further detailed investigations of subgroups in PAD patients are needed in order to elucidate how different stages of PAD affect mitochondrial function. The aim of this study was to investigate the severity‐dependent effects of PAD on mitochondrial respiration in patients with IC or chronic limb‐threatening ischemia.

By combining basic research to elucidate mitochondrial adaptability in patients with different PAD stages, this translational study aims to provide the foundation for a direct transfer of knowledge from the laboratory to the clinic.^20^

## 2 METHODS

### 2.1 Study population

In this single‐center study, all patients of the Department of Vascular Surgery were screened for study participation. Patients with symptomatic PAD were included consecutively. Exclusion criteria were age < 18 years, pregnancy, transplantation and emergency interventions in acute cases. Medical history and anthropometric parameters including sex, body mass index (BMI), age and cardiovascular risk factors (diabetes, arterial hypertension, smoking status and hyperlipidemia) were recorded. Diabetes mellitus was defined as fasting blood glucose ≥126 (≥7.0 mmol/L) and/or HbA1c in % ≥6.5 (≥48 mmol/mol haemoglobin). No distinction was made between diabetes mellitus type I and II. Pulse status, ankle brachial pressure index (ABPI) and presence of skin lesions on the lower extremities were assessed. Duplex ultrasound of the peripheral arteries was performed, and the atherosclerotic lesions were graded by severity and location (aortoiliac, femoropopliteal and distal lesions). The maximum walking distance was determined by a treadmill test at a continuous speed of 3 km per hour and an inclination of 0%. Two subgroups of PAD were defined: IC with indication for open or endovascular revascularization (Rutherford categories 2 and 3), and chronic limb threatening ischaemia (CLTI). CLTI comprised Rutherford categories 4 and 5, defined by chronic resting pain for at least 2 weeks and/or tissue loss related to PAD including non‐venous ulcerations or gangrene also lasting for at least 2 weeks being associated with hemodynamic parameters as defined by clinical guidelines.^5^ Twenty‐two control subjects with normal ABPI without walking distance restriction undergoing varicose vein surgery were included. Samples were collected once during non‐revascularizing procedures such as varicose vein surgery as well as from employees under local anaesthesia.

ABPI is not shown due to misleading values in the absence of compression in diabetics. Especially since the ABPI does not adequately explain the clinic as well as the myopathy of PAD patients.^9^, ^21^, ^22^

### 2.2 Muscle biopsy and permeabilization

Percutaneous biopsies of the vastus lateralis and gastrocnemius muscles of the affected leg were performed under general or local (lidocaine—without muscle infiltration to avoid potential effects of the medication on mitochondrial respiration) anaesthesia by using Bergstrom Muscle Biopsy Needles (Ø 4.0 mm, Dixon Surgical Instruments, Wickford, UK).^23^ Muscle tissue (~5 mg) was transferred into ice‐cold conservation fluid (BIOPS) (Appendix S1). After mechanical dissection, muscle fibres were chemically permeabilized by incubation in 2 mL of BIOPS containing 50 μg/mL saponine. After washing in a mitochondrial respiration medium (MiR06) (Appendix S1), around 1–3 mg wet weight was added to the respirometry chamber. The detailed procedure has been described elsewhere.^11^, ^12^, ^18^

### 2.3 Assessment of mitochondrial respiration

HRR was carried out by using an Oxygraph‐2k (Oroboros Instruments, Innsbruck, Austria) at 37°C as described before.^18^ The Oxygraph‐2k is composed of two respirometry chambers containing 2 mL MiR06 each. Oxygen concentration (μmol/L) and oxygen flux (pmol/[s mL]) were continuously recorded in real‐time using DatLab software (Datlab Version 7.3.0.3, Oroboros Instruments, Innsbruck, Austria). Mass‐specific mitochondrial respiration was set as oxygen consumption per second, per milligram of wet weight of muscle tissue (pmol/[s*mg]). The measurement starts with the addition of gaseous oxygen into the chambers to create a hyperoxygenated environment with >500 mM oxygen levels in order to avoid oxygen limitation of fibre respiration. A specific substrate, uncoupler, inhibitor titration (SUIT) protocol was performed to induce a sequence of respiratory states, as described before.^11^, ^12^, ^18^ Nonphosphorylating LEAK state, mitochondrial respiration in the absence of adenosine diphosphate (ADP), was recorded after titration of 2 mM malate and 0.2 mM octanoylcarnitine (MOct_L_). The addition of 5 mM ADP yields NADH electron transfer‐pathway state (N) oxidative phosphorylation (OXPHOS) capacity (MOct_P_), the addition of pyruvate (5 mM) plus succinate (10 mM) results in OXPHOS capacity (P) combining N‐linked and succinate (S)‐linked substrates. Titration of the protonophore carbonyl cyanide p‐(trifluoromethoxy) phenylhydrazone (FCCP; 0.05 mM steps) induces maximum oxygen flux and reveals electron transfer capacity (ET).

### 2.4 Citrate synthase activity

CSA was determined from frozen (stored at −80°C) homogenized muscle samples. After thawing the samples, they were transferred to CellLytic™ MT reagent (Sigma–Aldrich, St. Louis, MO, USA) and then mechanically homogenized using pestle and subsequently centrifuged. The QuantiPro™ BCA Assay Kit (Sigma–Aldrich, St. Louis, MO, USA) was used to determine the protein concentration of the protein‐rich supernatant. Subsequently, CSA was measured spectrophotometrically by using the Citrate Synthase Assay Kit (Sigma–Aldrich, St. Louis, MO, USA) at 412 nm and 25°C. To obtain the specific mitochondrial respiration expressed as pmol/(s*mg) per CSA, all results from the HRR mitochondrial respiration were normalized to CSA as a marker for mitochondrial content.^19^, ^24^

Schematic overview of the methods shown in Appendix S2.

### 2.5 GV ratio

Since we saw almost no differences in vastus lateralis muscle between IC and CLTI, we took vastus lateralis muscle as a baseline to minimize interindividual differences for better comparability of patients. Mitochondrial respiration and CSA in gastrocnemius muscle were compared to those in vastus lateralis muscle, and the ratios of gastrocnemius over vastus respiration were calculated for each participant (‘GV ratio’) for all respiratory states.

### 2.6 Statistics

Statistical analysis was carried out using SPSS 27 (IBM, Armonk, NY). Continuous data are given as median with interquartile range based on a non‐Gaussian distribution. Data are presented as box plots including median and lower/upper quartiles; whiskers denote 5% and 95% percentiles. Average age and sex frequency were calculated. Significance levels were determined using non‐parametric tests for non‐dependent samples (Mann–Whitney *U*‐test) as well as chi‐square for clinical parameters *p*‐values <0.05 were considered significant. Linear regression analysis was performed between age, active smoking and the collected parameters (Appendix S3 and S4).

### 2.7 Study approval

The institutions' medical ethical committee (Charité Ethics Committee, Universitätsmedizin Berlin) approved the protocol and the design of this study under proposal number EA4/139/16. All participants gave their written informed consent. All procedures were per the 1964 Helsinki Declaration and its latest amendment of 2013.

## 3 RESULTS

### 3.1 Baseline characteristics

This study included 65 PAD patients (CLTI: *n* = 18; IC: *n* = 47) and 22 non‐ischemic control persons without any evidence of PAD. Participants in the control group were significantly younger than those within the two PAD groups (47.5 years versus 67.0 [IC] and 72.0 [CLTI] years, *p* < 0.001). Linear regression analysis showed no correlation between age and the parameters collected from the IC and control group. In the CLTI group, there was a correlation between age and mitochondrial function, but not with mitochondrial content (Appendix S3). In PAD patients, cardiovascular risk factors (diabetes, smoking status, hypertension, hyperlipidemia) were more frequent than in individuals without PAD (*p* < 0.001). No significant differences were seen in the intake of relevant medications between IC and CLTI. The patients took cardiovascular medication more frequently than the control subjects. Non‐healing wounds, ulcers or tissue loss were observed in 14 of 18 CLTI patients, 7 of whom had pain at rest. PAD patients were more often active smokers compared to the control group. Linear regression analysis showed a correlation between active smoking and mitochondrial function only in the IC group (Appendix S4). Anthropometric and disease‐specific details are given in Table 1.

**TABLE 1:** Clinical parameters of patients and controls.

	Control	IC	CLTI	*p*‐value		
	(*n* = 22)	(*n* = 47)	(*n* = 18)	C/IC	C/CLTI	IC/CLTI
Age in years	47.5 (36.9–71.1)	67.0 (60.6–74.2)	72.0 (65.2–82.7)	<0.001	<0.001	0.241
Sex (%male)	12 (54.5)	38 (80.9)	13 (72.2)	0.023	0.251	0.449
Body mass index (BMI)	26.9 (24.9–31.7)	26.0 (22.6–29.4)	24.5 (20.1–29.5)	0.225	0.235	0.980
Diabetes	4 (18.2)	13 (27.7)	7 (38.9)	0.395	0.145	0.380
Active cigarette smoking	3 (13.6)	25 (53.2)	9 (50.0)	0.002	0.017	0.266
Hyperlipidemia	5 (23.8)	30 (63.8)	9 (50.0)	0.002	0.089	0.308
Any cardiovascular risk factor	7 (31.8)	40 (85.1)	15 (83.3)	<0.001	0.001	0.859
Permanent dialysis	1 (5.0)	3 (6.4)	3 (18.7)	0.827	0.242	0.200
Aortoiliac PAD		18 (38.3)	4 (22.2)			0.220
Femoropopliteal PAD		42 (89.4)	18 (100.0)			0.150
Distal PAD		5 (10.6)	6 (33.3)			0.029
No. of cardiovasc.risk factors	0 (0–2.25)	2.0 (1.0–3.0)	2.0 (1.0–3.0)	0.019	0.065	1.000
Statins	4 (18.2)	26 (55.3)	11 (61.1)	0.004	0.005	0.673
Antihypertensive drugs	7 (31.8)	35 (74.5)	13 (72.2)	0.001	0.011	0.854
Antiplatelet agents	4 (18.2)	40 (85.1)	14 (77.8)	<0.001	<0.001	0.481
Anticoagulation	0 (0)	11 (23.4)	6 (33.3)	0.013	0.003	0.415
Walking distance	No limitations	100	0	<0.001	<0.001	0.719

*Note*: Cardiovascular risk factors included diabetes mellitus, hyperlipidemia and smoking status, and are presented as number of risk factors present in patients; frequencies are presented as prevalence with portions in brackets; continuous data are expressed as median with interquartile range in brackets. Statistical analysis by Mann–Whitney test and chi‐square. A *p*‐value <0.05 is considered to be significant. *p*‐value shows comparison between control and patients (IC and CLTI).

Abbreviations: CLTI, chronic limb‐threatening ischemia; IC, intermittent claudication; PAD, peripheral arterial disease.

### 3.2 Mitochondrial content

CSA was lower in ischemic muscle compared to non‐ischemic controls. In the gastrocnemius muscle of CLTI patients, CSA was lower than in controls (265.5 vs. 340.6 nmol/min/mg, *p* = 0.031). In vastus muscles, CSA of both IC and CLTI were decreased compared to the control group (IC: 261.2 nmol/min/mg, *p* = 0.010; CLTI: 266.8 nmol/min/mg, *p* = 0.028). CSA in gastrocnemius (*p* = 0.344) and vastus (*p* = 0.616) was not different between different severity stages of PAD (IC versus CLTI) (Table 2; Appendix S5).

**TABLE 2:** Citrate synthase activity (CSA).

Muscle	*N*	CSA [nmol/min/mg protein]	*p*‐value
Gastrocnemius muscle			
Control (C)	15	340.6 (288.9–562.2)	C/IC: 0.121
IC	32	328.4 (231.3–385.9)	C/CLTI: 0.031
CLTI	11	265.5 (214.8–355.4)	IC/CLTI: 0.344
Vastus lateralis muscle			
Control (C)	11	311.7 (284.4–421.8)	C/IC: 0.010
IC	34	261.2 (196.4–314.9)	C/CLTI: 0.028
CLTI	11	266.8 (184.1–306.6)	IC/CLTI: 0.616

*Note*: CSA in gastrocnemius and vastus lateralis muscles, in controls, in patients with intermittent claudication (IC), and in chronic limb‐threatening ischemia (CLTI). Data are expressed as median with interquartile range in brackets. Statistical analysis by Mann–Whitney test. A *p*‐value <0.05 is considered to be significant.

### 3.3 Mitochondrial function

**FIGURE 1:**
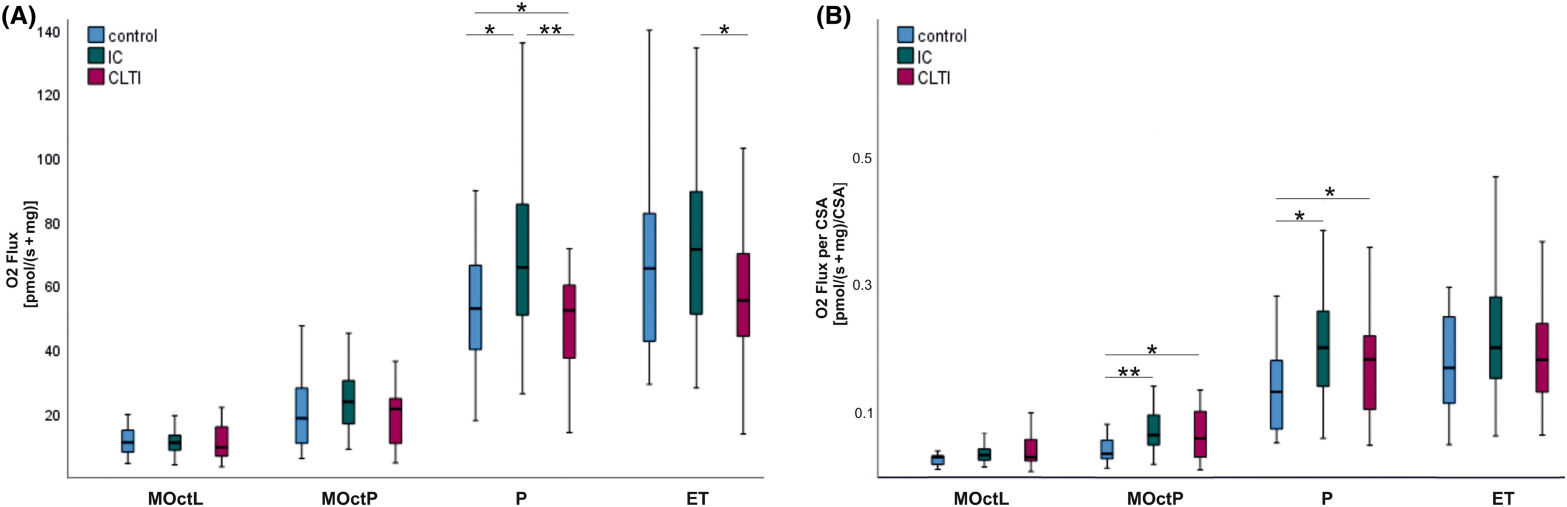
Respirometry results of gastrocnemius muscle from controls, intermittent claudication (IC) and chronic limb‐threatening ischemia (CLTI). (A) High‐resolution respirometry results are expressed as oxygen (O_2_) flux per mg wet weight (pmol/[s*mg]) and (B) normalized to citrate synthase activity (CSA) (pmol/[s*mg]/CSA). ET, maximal respiration after addition of FCCP; MOct_L_, maximal respiration after titration of malate and octanoyl; MOct_P_, maximal respiration after addition of ADP; P, maximal respiration after addition of pyruvate and succinate. Statistical analysis by Mann–Whitney test. *p < 0.05, **p < 0.01.

Mitochondrial respiration in gastrocnemius muscle of IC was higher compared to non‐ischemic controls and compared to CLTI for OXPHOS capacity (P) (IC vs. C: *p* = 0.045 and IC vs. CLTI: *p* = 0.008) as well as for ET (IC vs. CLTI *p* = 0.032) (Table 3; Figure 1A). In vastus muscles, no alterations of mitochondrial respiration in IC or CLTI compared to control were found except for a decrease in LEAK state (MOct_L_ *p* = 0.018) (Table 4).

**TABLE 3:** Mitochondrial respiration of gastrocnemius muscle.

	Control			IC			CLTI			*p*‐values		
	*N*	Median	IQR	*N*	Median	IQR	*N*	Median	IQR	C/IC	C/CLTI	IC/CLTI
GM‐Mitochondrial respiration (pmol/[s*mg])												
MOct_L_	20	11.0	7.4–15.0	43	11.0	8.2–13.7	18	9.5	6.3–14.4	0.848	0.943	0.740
MOct_P_	20	18.6	8.8–29.8	43	23.8	18.4–30.9	17	21.6	10.1–28.8	0.114	0.094	0.058
*P*	20	53.0	39.3–72.5	45	65.9	48.6–89.2	18	52.4	39.8–64.7	0.045	0.013	0.008
ET	20	65.6	40.0–85.4	45	71.5	49.4–96.3	18	55.6	42.5–75.6	0.327	0.093	0.032
GM‐Mitochondrial respiration (pmol/[s*mg]) per CSA												
MOct_L_	14	0.032	0.019–0.034	30	0.035	0.026–0.046	11	0.032	0.023–0.061	0.066	0.233	0.953
MOct_P_	14	0.037	0.028–0.065	29	0.067	0.050–0.103	11	0.061	0.030–0.117	0.007	0.030	0.388
*P*	14	0.134	0.076–0.199	31	0.203	0.140–0.271	11	0.185	0.080–0.243	0.017	0.046	0.203
ET	14	0.172	0.112–0.252	31	0.203	0.153–0.286	11	0.185	0.097–0.269	0.091	0.225	0.399

*Note*: Mitochondrial respiration of gastrocnemius muscle in the control group, in patients with intermittent claudication (IC), and chronic limb‐threatening ischemia (CLTI), as absolute values and normalized to citrate synthase activity (CSA). Statistical analysis by Mann–Whitney test. A *p*‐value <0.05 is considered to be significant.

Abbreviations: ET, maximal respiration after addition of FCCP; GM, gastrocnemius muscle; MOct_L_, maximal respiration after titration of malate and octanoyl; MOct_P_, maximal respiration after addition of ADP; P, maximal respiration after addition of pyruvate and succinate.

**TABLE 4:** Mitochondrial respiration of vastus lateralis muscle.

	Control			IC			CLTI			*p*‐values		
	*N*	Median	IQR	*N*	Median	IQR	*N*	Median	IQR	C/IC	C/CLTI	IC/CLTI
VML‐Mitochondrial respiration (pmol/[s*mg])												
MOct_L_	17	16.9	11.1–19.4	44	11.0	8.7–16.6	17	12.9	9.8–22.0	0.018	0.399	0.228
MOct_P_	17	22.3	14.5–31.9	44	22.4	15.5–28.6	17	21.0	12.7–25.8	0.834	0.438	0.412
P	17	63.7	47.2–67.9	45	60.9	42.9–69.9	17	54.1	40.1–62.5	0.906	0.235	0.163
ET	17	68.2	53.7–85.0	46	68.4	47.6–87.7	17	64.8	46.0–73.2	0.792	0.344	0.369
VML‐Mitochondrial respiration (pmol/[s*mg]) per CSA												
MOct_L_	10	0.051	0.031–0.067	32	0.046	0.032–0.056	11	0.046	0.031–0.064	0.734	0.951	0.956
MOct_P_	10	0.060	0.043–0.102	31	0.078	0.059–0.129	11	0.078	0.047–0.106	0.202	0.422	0.538
P	10	0.212	0.123–0.248	32	0.206	0.152–0.292	11	0.218	0.130–0.286	0.392	0.670	0.738
ET	10	0.213	0.149–0.273	33	0.231	0.168–0.386	11	0.241	0.170–0.367	0.301	0.610	0.903

*Note*: Mitochondrial respiration of vastus lateralis muscle in the control group, in patients with intermittent claudication (IC), and chronic limb‐threatening ischemia (CLTI), as absolute values and normalized to citrate synthase activity (CSA). Statistical analysis by Mann–Whitney test. A *p*‐value <0.05 is considered to be significant.

Abbreviations: ET, maximal respiration after addition of FCCP; MOct_L_, maximal respiration after titration of malate and octanoyl; MOct_P_, maximal respiration after addition of ADP; P, maximal respiration after addition of pyruvate and succinate; VML, vastus lateralis muscle.

### 3.4 Mitochondrial respiration normalized to CSA

Mitochondrial respiration normalized to CSA in gastrocnemius muscle was significantly increased for MOct_P_ (*p* = 0.007) and NS‐linked OXPHOS (*p* = 0.017) in patients with IC as well as for MOct_P_ (*p* = 0.030) and NS‐linked OXPHOS (*p* = 0.046) for the CLTI group when compared to controls (Table 3; Figure 1B).

In vastus muscle, there was no difference between claudicant, CLTI and control muscle samples regarding all defined respiratory states (Table 4).

### 3.5 Comparison of gastrocnemius and vastus muscle mitochondrial function—‘GV ratio’

**FIGURE 2:**
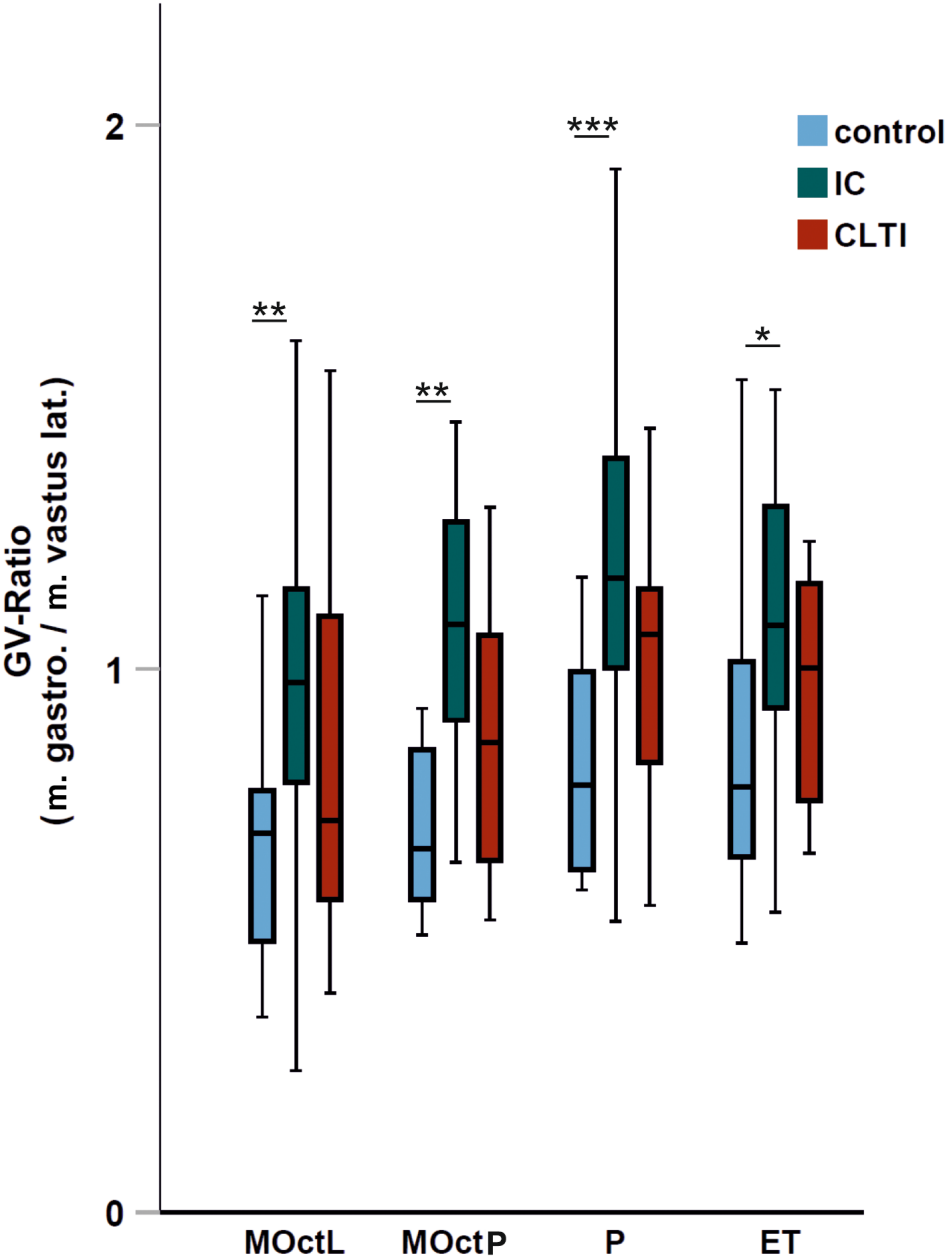
Ratio of respiration in gastrocnemius over vastus muscle (‘GV ratio’). In controls, intermittent claudication (IC) and chronic limb‐threatening ischemia (CLTI), for all respiratory states defined. ET, maximal respiration after addition of FCCP; MOctL, maximal respiration after titration of malate and octanoyl; MOctP, maximal respiration after addition of ADP; P, maximal respiration after addition of pyruvate and succinate. Statistical analysis by Mann–Whitney test. *p < 0.05, **p < 0.01, ***p < 0.001.

The GV ratios of mitochondrial respiration in IC compared to controls were significantly higher in all defined respiratory states. CLTI showed no difference from the controls (Table 5; Figure 2). No significant difference for CSA was found.

**TABLE 5:** Ratio of O_2_Flux in gastrocnemius muscle over vastus muscle (‘GV ratio’).

	Control			IC			CLTI			*p*‐value		
	*N*	Median	IQR	*N*	Median	IQR	*N*	Median	IQR	C/IC	C/CLTI	IC/CLTI
CSA	10	0.993	0.889–1.415	31	1.214	1.052–1.351	8	1.068	0.874–1.681	0.379	0.859	0.348
MOct_L_	13	0.697	0.485–0.817	30	0.974	0.789–1.161	10	0.721	0.541–1.209	0.002	0.535	0.134
MOct_P_	13	0.668	0.573–0.880	28	1.081	0.899–1.297	10	0.865	0.641–1.116	0.001	0.137	0.076
P	13	0.786	0.621–1.000	30	1.167	0.993–1.397	10	1.063	0.813–1.169	<0.001	0.063	0.065
ET	13	0.783	0.627–1.030	31	1.079	0.927–1.307	10	1.002	0.756–1.157	0.012	0.226	0.208

*Note*: Ratio of O_2_Flux in gastrocnemius muscle over vastus muscle (‘GV ratio’). Mitochondrial respiration (pmol/[s*mg]) expressed as median with IQR in brackets. Statistical analysis by Mann–Whitney test. A *p*‐value <0.05 is considered to be significant.

Abbreviations: CSA, citrate synthase activity; ET, maximal respiration after addition of FCCP; MOct_L_, maximal respiration after titration of malate and octanoyl; MOct_P_, maximal respiration after addition of ADP; P, maximal respiration after addition of pyruvate and succinate.

## 4 DISCUSSION

The aim of this study was to investigate mitochondrial respiration and content in skeletal muscle of patients suffering from PAD with different degrees of clinical severity. We were able to confirm our hypothesis that the impact of chronic ischemia on mitochondrial function is dependent on PAD severity.

Our study cohort comprised PAD patients with multiple comorbidities and an advanced age, which is in line with the natural history of the disease.^4^, ^25^

Unlike controls and IC patients, our CLTI patients exhibit a more or less sedentary lifestyle due to resting pain, and inability to walk because of ulcers or gangrene. Mitochondria are able to adapt to metabolic demands imposed by the individual level of physical activity, and inactivity decreases mitochondrial content.^26^, ^27^, ^28^ As marker for mitochondrial content, CSA was determined.^19^, ^24^ Compared to non‐ischemic controls, CSA was decreased in gastrocnemius muscle of IC and, to a larger degree, of CLTI (Table 2). In contrast, Ryan et al.^15^ as well as Pipinos et al.^13^ did not find any significant difference in CSA between more advanced PAD and controls. Other groups, however, found increased CSA in ischemic muscles compared to controls.^17^, ^29^ Park et al.^17^ investigated milder PAD Stages and Pipinos et al.^29^ investigated short medical history for a total of 14 weeks using staged ligation of common femoral and iliac arteries ligation in mice. Both indicate a shorter disease history with conceivable different adaptation mechanisms that require further investigation. Furthermore, it remains unclear whether CSA is a valid marker of mitochondrial content, particularly in patients with PAD.^24^

In our study, we found an increase in complex I‐ and complex II‐related respiration in the gastrocnemius muscle of IC patients over controls without PAD. This is in line with our previous findings.^11^, ^12^ This, however, was not true for the CLTI group (Table 3; Figure 1). Pipinos et al.^10^, ^13^ demonstrated a decrease in respiration in higher PAD stages compared to the control group. A closer look shows that these PAD stages are more similar to our CLTI group than to the IC group and could therefore confirm the lower respiration of the CLTI group. Unfortunately, there is also no comparative data available for milder PAD patients, which makes it difficult to classify the values. Similarly to our findings, Ryan et al.^15^ demonstrated a decreased respiration in CLTI patients compared to controls and IC patients, but no significant changes of respiration in the IC group, however, in their study only 7 IC patients were subjected to HRR. Park et al.^17^ investigated PAD patients with IC at microcirculation level. They showed a decrease in mitochondrial respiration in the PAD group. One limiting factor is the small number of patients (*n* = 10) and the difficulty in classifying the results due to insufficient patient data such as walking distance for explicit staging, as well as the reduced comparability in the absence of higher PAD stages and a missing normalization of respiration to mitochondrial content. One possible explanation for the contradictory results could be the selection of patients. Whereas in the current study, the IC group was defined as Rutherford categories 2 and 3 which undergo revascularization, in the study of Park et al.^17^ the samples are taken from patients presenting for an examination in symptomatic PAD without planned surgery. Our hypothesis is that higher PAD stages—requiring revascularization—is associated with an adaptive response in terms of decreasing mitochondrial content and increasing mitochondrial respiration, at least in milder stages such as IC. An inverse relationship could be explained by an earlier time point of the biopsy, at which no or another adaptation reaction could have taken place (Appendix S6). This is supported by the mouse study by Pipinos et al.,^29^ in which increased CSA with decreasing mitochondrial respiration was observed after a short course of the disease following ligation of the common femoral and iliac arteries over a period of only 14 weeks. In contrast, clinical arteriosclerosis presents as a gradual progression over several years, with PAD often progressing unnoticed.^30^, ^31^

In contrast, Hart et al.^16^ showed no differences of mitochondrial respiration and content between PAD patients and healthy control. Only increased mitochondrial‐derived ROS production, tumour necrosis factors, Interleukin 6, pro‐inflammatory and oxidative stress markers could be observed in the PAD group. They concluded that these processes precede impaired mitochondrial function. Comparability is limited due to the different selection of patients, as they only included 10 mild PAD stages (Fontaine IIa) without diabetes. The patients were therefore much healthier than in comparative studies.^10^, ^13^, ^15^, ^17^ Lindegaard Pedersen et al.^32^ showed preserved respiration in mild PAD stages without diabetics as well as decreased respiration in mild PAD with diabetics. This is in line with Park et al.^17^ and supports our conceivable course of PAD (Appendix S6). Since we included diabetes patients in all groups (Table 1), as did comparative studies,^15^, ^17^ and saw no significant difference in the distribution, this should not affect the validity of our study. Apart from falsely high, misleading ABPI measurements due to media sclerosis, so we do not present these in this study. Unfortunately, no alternative method, such as toe‐brachial pressures, was determined.

Further investigation of the different stages of PAD is therefore essential to better understand the exact course of the disease at the molecular level, in order to better classify the contrasting results. Improved understanding of the role of mitochondrial function in the pathogenesis of PAD may also open up further avenues for possible therapeutic strategies.

A promising approach to this was recently demonstrated by Ferrucci et al.^7^ with a multiomics analysis of PAD muscle biopsies. They analysed different mRNA as well as proteins from patients with mild PAD stages compared to controls and showed among other things reduced glucose metabolism, remodelling of the cytoskeleton and mismatch between transcription and translation of mitochondrial genes. Further examinations are required as only mild stages of PAD without diabetes were included.

Regarding the different degrees of mitochondriopathy in CLTI compared to IC and controls,^15^ as well as myopathy and neuropathy,^6^, ^33^, ^34^, ^35^, ^36^, ^37^, ^38^ more severe ischemia–reperfusion damage^39^, ^40^, ^41^, ^42^ is conceivable in higher PAD stages, causing decreased mitochondrial function^9^, ^10^, ^13^, ^22^, ^43^, ^44^ and could be an explanation of the limited adaptation of CLTI patients. Decreased physical activity in CLTI patients could also contribute to our findings. However, physical activity decrements have been shown to mainly influence mitochondrial content, as compared to respiration.^45^, ^46^ The inter‐individual range of variability in HRR values in patients is very high. As an intra‐individual control, we therefore used the vastus lateralis samples to normalize intra‐individual differences between patients and controls (Table 5). Furthermore, this ratio may help to diminish the bias of different levels of physical activity between individual patients. Supported by our data, the gastrocnemius muscle as the most distal end organ of PAD is more severely affected compared to vastus lateralis muscle. The GV‐ratios significantly increased in IC compared to controls and CLTI and supported our hypothesis of a limited mitochondrial adaption of higher PAD stages and higher adaptability of lower PAD stages.

Whether molecular differences in mitochondriopathy also exist after clinically successful revascularization between the different PAD stages needs further investigation.

As a limitation of our study, the control group was younger and exhibited less comorbidities than the two PAD groups. However, no correlation of mitochondrial respiration as well as content and age were found for the IC group. Only a correlation between mitochondrial respiration and age was found in the CLTI group (Appendix S3). This can be explained by the younger patients in the IC group, whereas the CLTI group is known to represent an older patient population.

Due to limited human biopsy material, only one marker of mitochondrial content (CSA) could be determined. Furthermore, we were, unfortunately, unable to determine markers for fission/fusion or ultrastructure such as mitochondrial morphology. However, it has been described that alterations in mitochondrial functions could be associated with morphology changes and fission/fusions in patients with PAD.^9^ Another possible limitation is that CSA was measured in lysates. However, it was not the same piece of tissue that was used for permeabilization for the HRR.

Medication usage may impact results. As expected, the controls differed from the patients with PAD in terms of frequency of cardiovascular medication use. However, there was no difference between the IC and CLTI groups. Thus, both groups could be compared with the control group and with each other under the same conditions.

In order to find out if the mitochondrial function is associated with other risk factors we did a linear regression between active cigarette smoking and the collected parameters (Appendix S4). Interestingly there was only a correlation between mitochondrial function but not mitochondrial content. Moreover, only in the IC group. It is already known, that smoking leads to oxidative stress and mitochondrial dysfunction.^47^ Why only the IC group shows a correlation remains unclear. A possible explanation could also be the reduced adaptability in the CLTI group compared to the IC group which needs to be investigated further.

In summary, we demonstrate a stage‐dependent effect of PAD on mitochondrial respiration and CSA. In IC, mitochondrial respiration is elevated compared to CLTI and non‐ischemic controls which may be interpreted as an adaptation to limited oxygen supply. When the degree of ischemia becomes more severe, the mitochondrial ability to adapt is limited.

## AUTHOR CONTRIBUTIONS

**Fiona Speichinger:** Conceptualization (equal); data curation (equal); formal analysis (equal); investigation (equal); methodology (equal); resources (equal); visualization (equal); writing – original draft (lead); writing – review and editing (equal). **Alexandra Gratl:** Conceptualization (equal); data curation (equal); investigation (equal); writing – review and editing (equal). **Ben Raude:** Data curation (equal); investigation (equal); writing – review and editing (equal). **Larissa Schawe:** Writing – review and editing (equal). **Jan Carstens:** Writing – review and editing (equal). **Nina A. Hering:** Writing – review and editing (equal). **Andreas Greiner:** Conceptualization (equal); writing – review and editing (equal). **Dominik Pesta:** Methodology (equal); resources (equal); writing – review and editing (equal). **Jan Paul Frese:** Conceptualization (equal); formal analysis (equal); methodology (equal); resources (equal); supervision (lead); visualization (equal); writing – review and editing (equal).

## CONFLICT OF INTEREST STATEMENT

The authors declare that they have no conflict of interest.

## Supporting information


Appendix S1.

## Data Availability

The dataset generated and analysed during the current study is available from the corresponding author upon reasonable request. All authors consent for publication.

## References

[1] Horváth L , Németh N , Fehér G , Kívés Z , Endrei D , Boncz I . Epidemiology of peripheral artery disease: narrative review. Life (Basel). 2022;12(7):1041. doi:10.3390/life12071041 35888129 PMC9320565

[2] Kengne AP , Echouffo‐Tcheugui JB . Differential burden of peripheral artery disease. Lancet Glob Health. 2019;7(8):e980‐e981. doi:10.1016/S2214-109X(19)30293-1 31303302

[3] Rutherford RB , Baker JD , Ernst C , et al. Recommended standards for reports dealing with lower extremity ischemia: revised version. J Vasc Surg. 1997;26(3):517‐538. doi:10.1016/s0741-5214(97)70045-4 9308598

[4] Baubeta Fridh E , Andersson M , Thuresson M , et al. Amputation rates, mortality, and pre‐operative comorbidities in patients revascularised for intermittent claudication or critical limb Ischaemia: a population based study. Eur J Vasc Endovasc Surg. 2017;54(4):480‐486. doi:10.1016/j.ejvs.2017.07.005 28797662

[5] Conte MS , Bradbury AW , Kolh P , et al. Global vascular guidelines on the management of chronic limb‐threatening ischemia. Eur J Vasc Endovasc Surg. 2019;58(1S):S1‐S109.e33. doi:10.1016/j.ejvs.2019.05.006 31182334 PMC8369495

[6] Kim K , Anderson EM , Scali ST , Ryan TE . Skeletal muscle mitochondrial dysfunction and oxidative stress in peripheral arterial disease: a unifying mechanism and therapeutic target. Antioxidants (Basel). 2020;9(12):1304. doi:10.3390/antiox9121304 33353218 PMC7766400

[7] Ferrucci L , Candia J , Ubaida‐Mohien C , et al. Transcriptomic and proteomic of gastrocnemius muscle in peripheral artery disease. Circ Res. 2023;132(11):1428‐1443. doi:10.1161/CIRCRESAHA.122.322325 37154037 PMC10213145

[8] Jain I , Oropeza BP , Huang NF . Multiomics analyses of peripheral artery disease muscle biopsies. Circ Res. 2023;132(11):1444‐1446. doi:10.1161/CIRCRESAHA.123.322913 37228238 PMC10275498

[9] Pipinos II , Judge AR , Selsby JT , et al. The myopathy of peripheral arterial occlusive disease: part 1. Functional and histomorphological changes and evidence for mitochondrial dysfunction. Vasc Endovasc Surg. 2007;41(6):481‐489. doi:10.1177/1538574407311106 18166628

[10] Pipinos II , Sharov VG , Shepard AD , et al. Abnormal mitochondrial respiration in skeletal muscle in patients with peripheral arterial disease. J Vasc Surg. 2003;38(4):827‐832. doi:10.1016/s0741-5214(03)00602-5 14560237

[11] Gratl A , Frese J , Speichinger F , et al. Regeneration of mitochondrial function in gastrocnemius muscle in peripheral arterial disease after successful revascularisation. Eur J Vasc Endovasc Surg. 2020;59(1):109‐115. doi:10.1016/j.ejvs.2019.08.011 31786105

[12] Gratl A , Pesta D , Gruber L , et al. The effect of revascularization on recovery of mitochondrial respiration in peripheral artery disease: a case control study. J Transl Med. 2021;19(1):244. doi:10.1186/s12967-021-02908-0 34088309 PMC8178834

[13] Pipinos II , Judge AR , Zhu Z , et al. Mitochondrial defects and oxidative damage in patients with peripheral arterial disease. Free Radic Biol Med. 2006;41(2):262‐269. doi:10.1016/j.freeradbiomed.2006.04.003 16814106

[14] Wallace DC . Mitochondrial defects in cardiomyopathy and neuromuscular disease. Am Heart J. 2000;139(2 Pt 3):S70‐S85. doi:10.1067/mhj.2000.103934 10650320

[15] Ryan TE , Yamaguchi DJ , Schmidt CA , et al. Extensive skeletal muscle cell mitochondriopathy distinguishes critical limb ischemia patients from claudicants. JCI Insight. 2018;3(21):e123235. doi:10.1172/jci.insight.123235 30385731 PMC6238738

[16] Hart CR , Layec G , Trinity JD , et al. Increased skeletal muscle mitochondrial free radical production in peripheral arterial disease despite preserved mitochondrial respiratory capacity. Exp Physiol. 2018;103(6):838‐850. doi:10.1113/EP086905 29604234 PMC7640985

[17] Park SY , Pekas EJ , Anderson CP , et al. Impaired microcirculatory function, mitochondrial respiration, and oxygen utilization in skeletal muscle of claudicating patients with peripheral artery disease. Am J Physiol Heart Circ Physiol. 2022;322(5):H867‐H879. doi:10.1152/ajpheart.00690.2021 35333113 PMC9018007

[18] Pesta D , Gnaiger E . High‐resolution respirometry: OXPHOS protocols for human cells and permeabilized fibers from small biopsies of human muscle. Methods Mol Biol. 2012;810:25‐58. doi:10.1007/978-1-61779-382-0_3 22057559

[19] Larsen S , Nielsen J , Hansen CN , et al. Biomarkers of mitochondrial content in skeletal muscle of healthy young human subjects. J Physiol. 2012;590(14):3349‐3360. doi:10.1113/jphysiol.2012.230185 22586215 PMC3459047

[20] Rubio DM , Schoenbaum EE , Lee LS , et al. Defining translational research: implications for training. Acad Med. 2010;85(3):470‐475. doi:10.1097/ACM.0b013e3181ccd618 20182120 PMC2829707

[21] McDermott MM , Ferrucci L , Gonzalez‐Freire M , et al. Skeletal muscle pathology in peripheral artery disease: a brief review. Arterioscler Thromb Vasc Biol. 2020;40(11):2577‐2585. doi:10.1161/ATVBAHA.120.313831 32938218 PMC9571495

[22] Pipinos II , Judge AR , Selsby JT , et al. The myopathy of peripheral arterial occlusive disease: part 2. Oxidative stress, neuropathy, and shift in muscle fiber type. Vasc Endovascular Surg. 2008;42(2):101‐112. doi:10.1177/1538574408315995 18390972 PMC12282609

[23] Bergstrom J . Percutaneous needle biopsy of skeletal muscle in physiological and clinical research. Scand J Clin Lab Invest. 1975;35(7):609‐616. 1108172

[24] Groennebaek T , Billeskov TB , Schytz CT , et al. Mitochondrial structure and function in the metabolic myopathy accompanying patients with critical limb ischemia. Cell. 2020;9(3):570. doi:10.3390/cells9030570 PMC7140415 32121096

[25] Fowkes FGR , Rudan D , Rudan I , et al. Comparison of global estimates of prevalence and risk factors for peripheral artery disease in 2000 and 2010: a systematic review and analysis. Lancet. 2013;382(9901):1329‐1340. doi:10.1016/S0140-6736(13)61249-0 23915883

[26] Holloszy JO , Coyle EF . Adaptations of skeletal muscle to endurance exercise and their metabolic consequences. J Appl Physiol Respir Environ Exerc Physiol. 1984;56(4):831‐838. doi:10.1152/jappl.1984.56.4.831 6373687

[27] Wibom R , Hultman E , Johansson M , Matherei K , Constantin‐Teodosiu D , Schantz PG . Adaptation of mitochondrial ATP production in human skeletal muscle to endurance training and detraining. J Appl Physiol (1985). 1992;73(5):2004‐2010. doi:10.1152/jappl.1992.73.5.2004 1474078

[28] Pesta D , Hoppel F , Macek C , et al. Similar qualitative and quantitative changes of mitochondrial respiration following strength and endurance training in normoxia and hypoxia in sedentary humans. Am J Physiol Regul Integr Comp Physiol. 2011;301(4):R1078‐R1087. doi:10.1152/ajpregu.00285.2011 21775647

[29] Pipinos II , Swanson SA , Zhu Z , et al. Chronically ischemic mouse skeletal muscle exhibits myopathy in association with mitochondrial dysfunction and oxidative damage. Am J Physiol Regul Integr Comp Physiol. 2008;295(1):R290‐R296. doi:10.1152/ajpregu.90374.2008 18480238 PMC2494805

[30] Marso SP , Hiatt WR . Peripheral arterial disease in patients with diabetes. J Am Coll Cardiol. 2006;47(5):921‐929. doi:10.1016/j.jacc.2005.09.065 16516072

[31] McDermott MM . Lower extremity manifestations of peripheral artery disease: the pathophysiologic and functional implications of leg ischemia. Circ Res. 2015;116(9):1540‐1550. doi:10.1161/CIRCRESAHA.114.303517 25908727 PMC4410164

[32] Lindegaard Pedersen B , Bækgaard N , Quistorff B . Mitochondrial dysfunction in calf muscles of patients with combined peripheral arterial disease and diabetes type 2. Int Angiol. 2017;36(5):482‐495. doi:10.23736/S0392-9590.17.03824-X 28291304

[33] Regensteiner JG , Wolfel EE , Brass EP , et al. Chronic changes in skeletal muscle histology and function in peripheral arterial disease. Circulation. 1993;87(2):413‐421. doi:10.1161/01.cir.87.2.413 8425290

[34] McGuigan MR , Bronks R , Newton RU , et al. Muscle fiber characteristics in patients with peripheral arterial disease. Med Sci Sports Exerc. 2001;33(12):2016‐2021. doi:10.1097/00005768-200112000-00007 11740293

[35] Tuomisto TT , Rissanen TT , Vajanto I , Korkeela A , Rutanen J , Ylä‐Herttuala S . HIF‐VEGF‐VEGFR‐2, TNF‐alpha and IGF pathways are upregulated in critical human skeletal muscle ischemia as studied with DNA array. Atherosclerosis. 2004;174(1):111‐120. doi:10.1016/j.atherosclerosis.2004.01.015 15135259

[36] Robbins JL , Jones WS , Duscha BD , et al. Relationship between leg muscle capillary density and peak hyperemic blood flow with endurance capacity in peripheral artery disease. J Appl Physiol (1985). 2011;111(1):81‐86. doi:10.1152/japplphysiol.00141.2011 21512146 PMC3137528

[37] Weiss DJ , Casale GP , Koutakis P , et al. Oxidative damage and myofiber degeneration in the gastrocnemius of patients with peripheral arterial disease. J Transl Med. 2013;11:230. doi:10.1186/1479-5876-11-230 24067235 PMC3849592

[38] Koutakis P , Myers SA , Cluff K , et al. Abnormal myofiber morphology and limb dysfunction in claudication. J Surg Res. 2015;196(1):172‐179. doi:10.1016/j.jss.2015.02.011 25791828 PMC4512658

[39] Pan J , Konstas AA , Bateman B , Ortolano GA , Pile‐Spellman J . Reperfusion injury following cerebral ischemia: pathophysiology, MR imaging, and potential therapies. Neuroradiology. 2007;49(2):93‐102. doi:10.1007/s00234-006-0183-z 17177065 PMC1786189

[40] Murphy E , Steenbergen C . Mechanisms underlying acute protection from cardiac ischemia‐reperfusion injury. Physiol Rev. 2008;88(2):581‐609. doi:10.1152/physrev.00024.2007 18391174 PMC3199571

[41] Jaeschke H , Woolbright BL . Current strategies to minimize hepatic ischemia‐reperfusion injury by targeting reactive oxygen species. Transplant Rev (Orlando). 2012;26(2):103‐114. doi:10.1016/j.trre.2011.10.006 22459037 PMC3575198

[42] Bernardi P . The mitochondrial permeability transition pore: a mystery solved? Front Physiol. 2013;4:95. doi:10.3389/fphys.2013.00095 23675351 PMC3650560

[43] Paradis S , Charles AL , Meyer A , et al. Chronology of mitochondrial and cellular events during skeletal muscle ischemia‐reperfusion. Am J Physiol Cell Physiol. 2016;310(11):C968‐C982. doi:10.1152/ajpcell.00356.2015 27076618 PMC4935201

[44] Rontoyanni VG , Nunez Lopez O , Fankhauser GT , Cheema ZF , Rasmussen BB , Porter C . Mitochondrial bioenergetics in the metabolic myopathy accompanying peripheral artery disease. Front Physiol. 2017;8:141. doi:10.3389/fphys.2017.00141 28348531 PMC5346567

[45] Van Schaardenburgh M , Wohlwend M , Rognmo Ø , Mattsson E . Calf raise exercise increases walking performance in patients with intermittent claudication. J Vasc Surg. 2017;65(5):1473‐1482. doi:10.1016/j.jvs.2016.12.106 28285932

[46] van Schaardenburgh M , Wohlwend M , Rognmo Ø , Mattsson EJR . Exercise in claudicants increase or decrease walking ability and the response relates to mitochondrial function. J Transl Med. 2017;15(1):130. doi:10.1186/s12967-017-1232-6 28592294 PMC5463401

[47] Dikalov S , Itani H , Richmond B , et al. Tobacco smoking induces cardiovascular mitochondrial oxidative stress, promotes endothelial dysfunction, and enhances hypertension. Am J Physiol Heart Circ Physiol. 2019;316(3):H639‐H646. doi:10.1152/ajpheart.00595.2018 30608177 PMC6459311

