# Supplementary figures and images for: Mitochondrial respiration in peripheral arterial disease depends on stage severity
Source: J Cell Mol Med. 2024 Mar 27;28(8):e18126. doi: 10.1111/jcmm.18126 (PMC10967142; doi:10.1111/jcmm.18126)

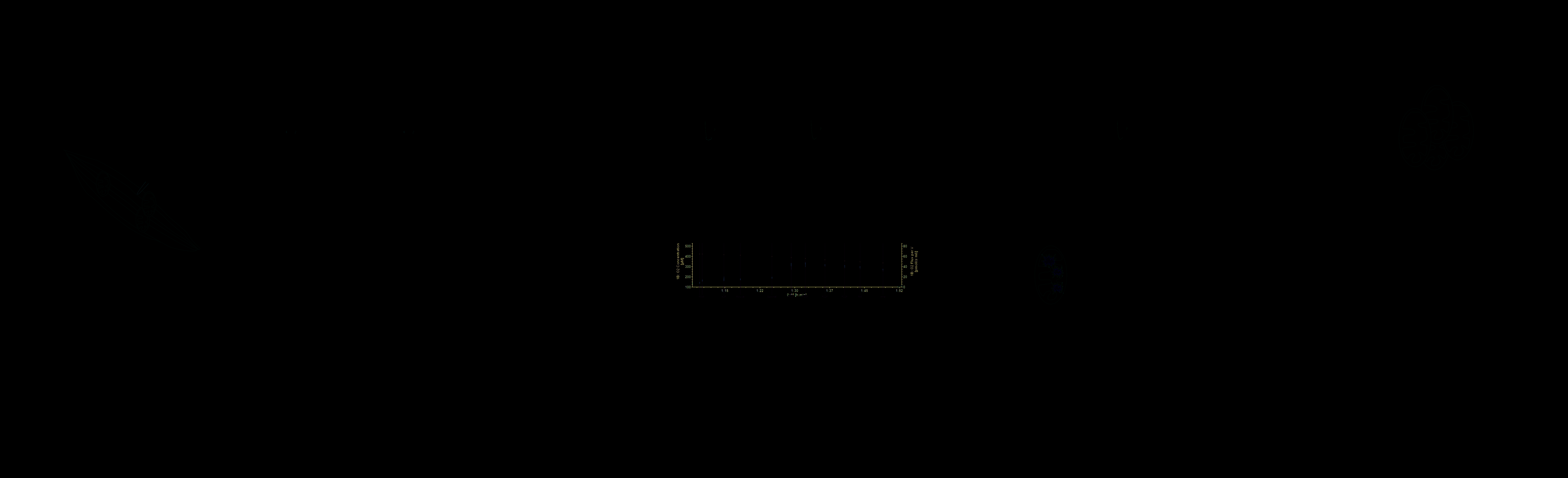

Supplement: Supplementary file 1 — Appendix S1. [file JCMM-28-e18126-s001.zip › Fig.Supp.2.tif]

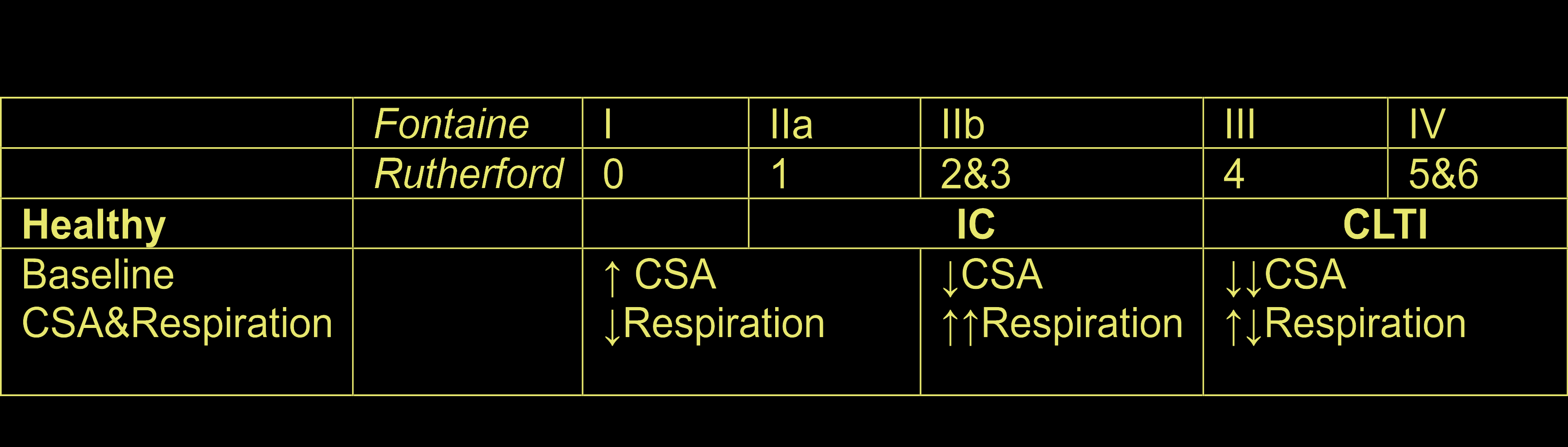

Supplement: Supplementary file 1 — Appendix S1. [file JCMM-28-e18126-s001.zip › Fig.Supp.6.tif]
