# Supplementary material for: Mitochondrial respiration in peripheral arterial disease depends on stage severity
Source: J Cell Mol Med. 2024 Mar 27;28(8):e18126. doi: 10.1111/jcmm.18126 (PMC10967142; doi:10.1111/jcmm.18126)
Supplement: Supplementary file 4 [file JCMM-28-e18126-s001.zip › jcmm18126-sup-0001-AppendixS1.odt]

Supplementary information

Supplement 1

BIOPS

10 mM Ca-EGTA buffer, 0.1µM free calcium, 20 mM imidazole, 20mM taurine, 50 mM 2-(N-morpholino)esthane-sulfonic acid hydrate, 0.5 mM dithiothreitol, 6.25mM MgCl₂, 5.77 ATP, 15 mM phosphocreatine (pH 7.1)

MiR06

110 mM D-sucrose, 60 mM K+-lactobionate, 0.5 mM EGTA, 3 mM MgCl₂, 20 mM taurine, 10 mM KH₂PO4, 20 mM HEPES, 1g/l bovine serum albumin, 280 U/ml catalase

Supplement 2

Time-dependent schematic representation of the methods. Determination of Citrate synthase activity (CSA) and High-resolution respirometry (HRR). ww = wet weight.

Supplement 3

| Supplement 3. Linear Regression Analysis_Age |
| --- |
| Group-muscle |  | R | R² | p-value |
| C-GM | CSA | 0.326 | 0.106 | 0.235 |
| MOctL | 0.537 | 0.289 | 0.015 |
| MOctP | 0.187 | 0.035 | 0.431 |
| P | 0.169 | 0.028 | 0.477 |
| ET | 0.175 | 0.030 | 0.462 |
| C-VLM | CSA | 0.141 | 0.020 | 0.680 |
| MOctL | 0.082 | 0.007 | 0.754 |
| MOctP | 0.004 | 0.000 | 0.987 |
| P | 0.141 | 0.020 | 0.588 |
| ET | 0.254 | 0.065 | 0.324 |
| IC-GM | CSA | 0.076 | 0.006 | 0.678 |
| MOctL | 0.105 | 0.011 | 0.503 |
| MOctP | 0.257 | 0.066 | 0.096 |
| P | 0.219 | 0.048 | 0.148 |
| ET | 0.135 | 0.018 | 0.377 |
| IC-VLM | CSA | 0.296 | 0.088 | 0.089 |
| MOctL | 0.257 | 0.066 | 0.092 |
| MOctP | 0.051 | 0.003 | 0.744 |
| P | 0.097 | 0.009 | 0.526 |
| ET | 0.060 | 0.004 | 0.692 |
| CLTI-GM | CSA | 0.410 | 0.168 | 0.211 |
| MOctL | 0.669 | 0.448 | 0.002 |
| MOctP | 0.600 | 0.360 | 0.011 |
| P | 0.509 | 0.259 | 0.031 |
| ET | 0.561 | 0.314 | 0.015 |
| CLTI-VLM | CSA | 0.259 | 0.067 | 0.442 |
| MOctL | 0.497 | 0.247 | 0.042 |
| MOctP | 0.780 | 0.680 | <0.001 |
| P | 0.671 | 0.450 | 0.003 |
| ET | 0.693 | 0.480 | 0.002 |

Linear regression analysis of the measured parameters with age in Control, IC and CLTI patients. C: Control. IC: intermittent claudication. CLTI: chronic limb threatening ischemia. GM: gastrocnemius muscle. VLM: vastus lateralis muscle. A p-value <0.05 is considered to be significant. 

Supplement 4

| Supplement 4. Linear Regression Analysis_active smoking |
| --- |
| Group-muscle |  | R | R² | p-value |
| C-GM | CSA | 0.075 | 0.006 | 0.790 |
| MOctL | 0.259 | 0.067 | 0.270 |
| MOctP | 0.021 | 0.000 | 0.929 |
| P | 0.254 | 0.065 | 0.279 |
| ET | 0.022 | 0.000 | 0.927 |
| C-VLM | CSA | 0.297 | 0.088 | 0.376 |
| MOctL | 0.207 | 0.043 | 0.426 |
| MOctP | 0.169 | 0.028 | 0.517 |
| P | 0.503 | 0.253 | 0.040 |
| ET | 0.273 | 0.074 | 0.290 |
| IC-GM | CSA | 0.154 | 0.024 | 0.399 |
| MOctL | 0.204 | 0.042 | 0.190 |
| MOctP | 0.467 | 0.218 | 0.002 |
| P | 0.338 | 0.114 | 0.023 |
| ET | 0.446 | 0.199 | 0.002 |
| IC-VLM | CSA | 0.230 | 0.053 | 0.192 |
| MOctL | 0.260 | 0.068 | 0.088 |
| MOctP | 0.570 | 0.325 | <0.001 |
| P | 0.423 | 0.179 | 0.004 |
| ET | 0.553 | 0.306 | <0.001 |
| CLTI-GM | CSA | 0.156 | 0.024 | 0.647 |
| MOctL | 0.209 | 0.044 | 0.405 |
| MOctP | 0.082 | 0.007 | 0.755 |
| P | 0.004 | 0.000 | 0.987 |
| ET | 0.093 | 0.009 | 0.713 |
| CLTI-VLM | CSA | 0.086 | 0.007 | 0.802 |
| MOctL | 0.286 | 0.082 | 0.267 |
| MOctP | 0.008 | 0.000 | 0.975 |
| P | 0.322 | 0.103 | 0.208 |
| ET | 0.150 | 0.023 | 0.565 |

Linear regression analysis of the measured parameters with active smoking in Control, IC and CLTI patients. IC: intermittent claudication. CLTI: chronic limb threatening ischemia. GM: gastrocnemius muscle. VLM: vastus lateralis muscle.

Supplement 5

 Citrate synthase activity (CSA). (A) Gastrocnemius, and (B) vastus lateralis muscles, in controls, in patients with intermittent claudication (IC), and in chronic limb threatening ischemia (CLTI). Statistical analysis by Mann-Whitney test. *p < 0.05.

Supplement 6

Conceivable course of PAD with regard to mitochondrial content (Citrate synthase activity CSA) and mitochondrial respiration in relation to the different stages of PAD ad classification systems. Intermittent claudication (IC), chronic limb threatening ischemia (CLTI).
